# Expression and Function of WNT6: From Development to Disease

**DOI:** 10.3389/fcell.2020.558155

**Published:** 2020-12-09

**Authors:** Ming Wei, Congmin Zhang, Yujia Tian, Xiaohui Du, Qi Wang, Hui Zhao

**Affiliations:** ^1^Department of Respiratory Medicine, The Second Hospital of Dalian Medical University, Dalian, China; ^2^Department of Scientific Research Center, The Second Hospital of Dalian Medical University, Dalian, China; ^3^The Health Check Up Center, The Second Hospital of Dalian Medical University, Dalian, China

**Keywords:** disease, development, differentiation, organ formation, Wnt6

## Abstract

WNT family member 6 (WNT6) is a member of the highly conserved WNT protein family. It plays an essential role in the normal development process, not only in embryonic morphogenesis, but also in post-natal homeostasis. WNT6 functions in mice and humans. This review summarizes the current findings on the biological functions of WNT6, describing its involvement in regulating embryogenesis, decidualization, and organ development. Aberrant WNT6 signaling is related to various pathologies, such as promoting cancer development, lung tuberculosis, and kidney fibrosis and improving the symptoms of Rett syndrome (RTT). Thus, due to its various functions, WNT6 has great potential for in-depth research. This work not only describes the signaling mechanism and function of WNT6 under physiological and pathological conditions, but also provides a theoretical basis for targeted therapy.

## Introduction

**FIGURE 1:**
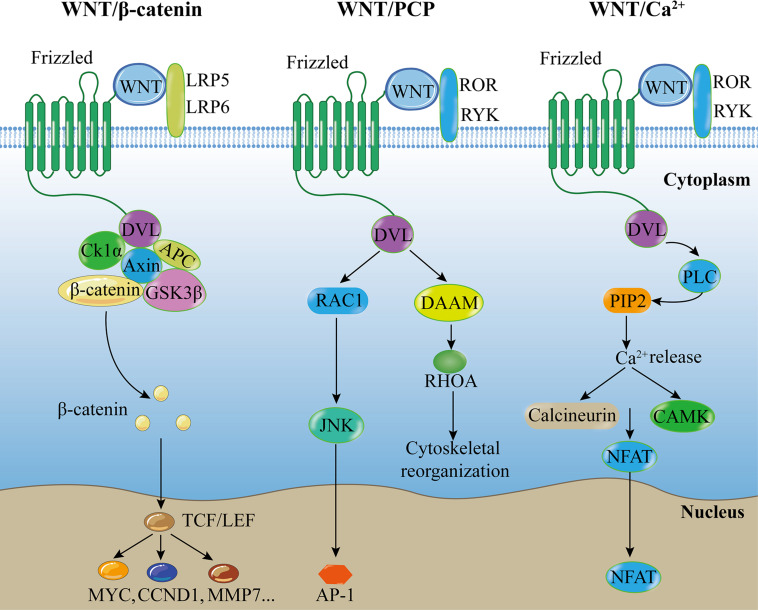
Canonical and non-canonical WNT pathways. Canonical WNT pathway: Extracellular WNT ligands bind to FZD and LRP5/6 on the cell membrane, and recruit DVL protein to promote dissociation of the β-catenin destruction complex, followed by accumulation of β-catenin, which translocates to the nucleus to interact with target downstream signaling molecules, activating intracellular signaling cascades. WNT/PCP pathway: Extracellular non-canonical WNT ligands bind to FZD receptor and/or ROR/RYK co-receptors at the cell membrane, and recruit DVL protein to promote downstream effector components, such as RAC and RHOA signaling cascades, regulating tissue polarity and cell movement. WNT/Ca^2+^ pathway: Extracellular WNT ligands bind to FZD receptor and/or ROR/RYK co-receptors at the cell membrane, and recruit DVL protein to promote PLC, leading to the formation of IP3 and DAG from PIP2, resulting in intracellular Ca^2+^ accumulation. Ca^2+^ can activate CAMK and NFAT transcription factor to regulate downstream cascades. Abbreviations: PCP, planar cell polarity; LRP, low-density lipoprotein receptor-related proteins; ROR, receptor tyrosine kinase-like orphan receptor; RYK, receptor-like tyrosine kinase; DVL, disheveled; CK1α, Casein Kinase-1α; APC, adenomatous polyposis coli; GSK3β, glycogen synthase kinase-3β; DAAM, disheveled associated activator of morphogenesis; PLC, phospholipase C; PIP2, phospholipid phosphatidylinositol 4,5-bisphosphate; JNK, Jun-N-terminal kinase; RHOA, ras homolog family member A; CAMK, calmodulin-dependent protein kinase; NFAT, nuclear factor of activated T cells; TCF/LEF, T-cell factor/lymphoid enhancer factor; CCND1, Cyclin D1; MMP7, matrix metallopeptidase 7; AP-1, activator protein 1.

WNT family member 6 (WNT6), a member of the Wingless/integrase 1 (WNT) family comprising at least 19 members in mammals, is a secreted glycoprotein. The WNT signaling family is highly conserved and in several species play essential roles in various physiological processes during which it is either stimulated or inhibited. WNT signals mediate various cellular functions by binding to different receptors on the cell membrane including 10 members of human Frizzled (FZD) receptors, low-density lipoprotein receptor-related proteins 5 and 6 (LRP5/6) receptor, and many non-class FZD receptors (Baarsma et al., 2013). The WNT signaling pathways are classified into the canonical WNT or WNT/β-catenin pathway, and the non-canonical WNT or β-catenin-independent pathways (Figure 1).

The WNT/β-catenin pathway is stimulated by an extracellular WNT ligand that binds to a FZD receptor and a LRP5/6 co-receptor (FZD and LRP5/6) on the cell membrane and recruits disheveled (DVL) proteins to promote the dissociation of the β-catenin destruction complex, leading to an intracellular signaling cascade. The β-catenin destruction complex consists of the scaffold protein Axin, Adenomatous Polyposis Coli (APC), Casein Kinase-1 (CK-1), and Glycogen Synthase Kinase-3β (GSK-3β). In the absence of WNT signaling, the β-catenin destruction complex phosphorylates β-catenin, leading to subsequent proteasomal degradation of the protein. However, in the presence of WNT signaling, WNTs inactivate the β-catenin destruction complex and prevent β-catenin degradation, thereby allowing β-catenin accumulation in the cytoplasm and translocation into the nucleus. Then β-catenin binds to the T-cell factor (TCF)/lymphoid enhancer factor (LEF) family of transcription factors, mediating the activation of downstream targets (Willert and Jones, 2006; MacDonald et al., 2009; Baarsma et al., 2013) such as Cyclin D1 (CCND1, a G1 phase cyclin), c-Myc (a proto-oncogene), and matrix metallopeptidase 7 (MMP7, a Zn2 + -dependent proteolytic enzyme) (Li et al., 2019).

In the β-catenin-independent WNT pathways, most WNT ligands bind to FZD receptors, whereas others interact with receptor tyrosine kinases of the ROR and RYK families (Angers and Moon, 2009; Clevers et al., 2014; Schulte, 2015; Shi et al., 2017). The β-catenin-independent WNT pathways are primarily classified into the WNT/Planar cell polarity (PCP) and WNT/Ca^2+^ pathways. The WNT/PCP pathway is associated with the downstream activation of c- Jun N-terminal kinase (JNK) and ras homolog family member A (RhoA) (Hwang and Kelly, 2012; Baarsma et al., 2013). The WNT/PCP signaling pathway regulates cell adhesion, migration, and polarity (Golenia et al., 2017; Shi et al., 2017; Villasenor et al., 2017). Conversely, the WNT/Ca^2+^ pathway is associated with the activation of phospholipase C (PLC) and leads to the formation of inositol 1,4,5-triphosphate (IP3) and 1,2 diacylglycerol (DAG), thereby increasing Ca^2+^ level and activating downstream targets. The WNT/Ca^2+^ pathway is associated with muscle contraction, gene transcription, and enzyme activation (Foulquier et al., 2018) and activates both β-catenin-dependent and β-catenin-independent pathways (Villasenor et al., 2017).

WNT6, a WNT family member that is highly conserved in various species, mainly considered to be a member of the β-catenin-dependent WNT signaling pathway (Krawetz and Kelly, 2008; Hwang and Kelly, 2012). However, it also functions through the β-catenin-independent pathways (Li et al., 2014; Schmeckpeper et al., 2015). WNT6 plays a critical role in the early development of embryos, and promotes the normal formation of various organs during the late development phase. It regulates post-natal tissue homeostasis and pathological disorders throughout the lifespan of organisms.

Because WNT6 is closely associated with many diseases, understanding its biology is important. Our review discusses the functions of WNT6 and its various signaling pathways, identified through studies of various *in vivo* mouse models, *in vitro* mouse and human cell systems, and clinical data in humans.

## Basic Information on WNT6

WNT signaling components are highly conserved with respect to both structure and function (Lavery et al., 2008; Foulquier et al., 2018). The term WNT is a fusion of the wingless (Wg) gene and the homologous vertebrate oncogene, intergrase-1 (int-1) (Baarsma et al., 2013). WNT ligands are a family of secreted glycoproteins consisting of at least 19 members in mammals (Cawthorn et al., 2012). WNT6, an evolutionarily conserved morphogenetic factor, belongs to the WNT family of secreted, hydrophobic glycoprotein (Yuan et al., 2013). The *wnt6* gene is located in the 2q35 region of the human chromosome, and encodes a 365 amino-acid polypeptide with an N-terminal signal peptide, a WNT core domain and a RGD motif. *Wnt6* is fused with *wnt10a* in a head-to-tail manner, with an interval less than 7.0 kb (Kirikoshi et al., 2001); therefore, WNT6 and WNT10a have some similar functions. For example, both WNT6 and WNT10a inhibit adipogenesis and stimulate osteoblastogenesis (Cawthorn et al., 2012). However, among the WNT proteins, WNT1 is the most homologous to WNT6 in the human genome (Kirikoshi et al., 2001); They have synergistic effects in mice (Otto et al., 2008; Hitchins et al., 2013; Huang et al., 2019) and antagonistic effects in humans and mice (Otto et al., 2008; Scheller et al., 2008; Hitchins et al., 2013; Li et al., 2014) at different time points.

**FIGURE 2:**
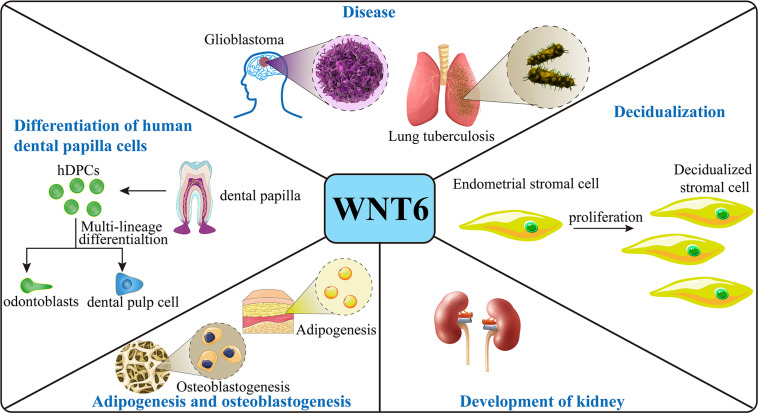
Function of WNT6. In the mouse embryo, WNT6 is expressed and promote stromal cell proliferation during decidualization and induces pre-tubular aggregation in the mesenchyme during formation of the renal tubule. WNT6 also prevents adipogenesis and stimulates osteoblastogenesis in mice. With respect to disease, in mice, loss of WNT6 correlates with the development of kidney fibrosis, and WNT6 can maintain the integrity of epithelium. WNT6 expression promotes the development of lung tuberculosis and GBM. In human cell models, WNT6 expression promotes the differentiation and migration of hDPCs and promotes the formation and development of tumors.

WNT6 plays a critical role in several aspects of mouse embryonic development. Furthermore, WNT6 expression is crucial for post-natal tissue homeostasis as well as pathological disorders during the lifespan of mice and humans (Figure 2).

## Functions of WNT6

### Decidualization

In both mice and humans, with the initiation of attachment, endometrial stromal cells surrounding the implanting blastocysts undergo decidualization to embed the embryo into the antimesometrial endometrial bed. The process of decidualization includes stromal cell proliferation and stromal decidual transformation with polyploidization. *Wnt6* mRNA is overexpressed in the stromal cells during decidualization, which occurs between days 5 and 8 of pregnancy. Mice without WNT6 expression have compromised term pregnancy with substantially reduced litter size, demonstrating that WNT6 signaling is essential for normal pregnancy (Wang et al., 2013). WNT6 deficiency markedly blocks stromal cell proliferation by prolonging the stromal cell cycle, which might be associated with decreased cyclin B1 (CCNB1), a key regulatory protein controlling cell cycle progression. Cyclin B1 is also highly expressed in the proliferative and secretory endometrium in humans (Tang et al., 2009; Wang et al., 2013). It is possible that WNT6 signaling plays a similar role during decidualization in humans. However, the loss of WNT6 has no significant effect on the differentiation of stromal cells into polyploidy decidual cells, another main process of decidualization (Wang et al., 2013). In summary, WNT6 plays a critical role in stromal cell proliferation during decidualization, thereby affecting normal embryonic development in mice, indicating that WNT6 begins to function during embryo formation, at least in mice.

### Kidney Development

Renal development is initiated following the formation of the epithelial ureter bud from the nephric duct and invasion of the metanephric blastema in mice (Beaton et al., 2016). Among them, the ureter tip region is the source of the primary inducer of kidney tubule differentiation (Itaranta et al., 2002). In the mouse embryo, *wnt6* mRNA is expressed in the early ureter bud and throughout the invading ureter bud when the epithelial ureter bud grows into the nephric mesenchyme. It is also expressed in the branching ureter tips where new branches are formed during the early stage of kidney development in mouse embryo. However, *wnt6* mRNA expression is reduced during growth of the ureter, possibly because of the maturing nephrogenic mesenchyme that releases inhibitory factors to decrease WNT6 expression. WNT6 protein aids in the establishment of tubular epithelial structures, increases the number of differentiated tubules, and plays a role in further maturation. Simultaneously, the expression of paired box gene (Pax2), Pax8, and E-cadherin is markedly increased in the mesenchyme (Itaranta et al., 2002). Pax2 is a marker of induction (Dressler et al., 1990) and Pax8 is a marker of pre-tubular cell aggregation (Plachov et al., 1990). All genes are markers for pre-tubular aggregates. These results indicate that WNT6 induces pre-tubular aggregation in the mesenchyme by upregulating these genes. However, WNT6 cannot induce ureter tubule growth and branching (Lin et al., 2001; Itaranta et al., 2002). In summary, WNT6 expression stimulates the interaction between the epithelium and mesenchyme and regulates mesenchyme development to induce kidney tubule formation rather than auto-regulating the epithelium (Itaranta et al., 2002).

### Differentiation of Human Dental Papilla Cells

Human dental papilla cells (hDPCs), the precursors of odontoblasts in human dental papilla tissue, have the potential to differentiate into odontoblasts and dental pulp cells (Huang et al., 2008), indicating that they play a critical role in tooth formation and development. WNT6 has been detected in the oral and dental epithelium and in the primary and secondary enamel knots, and low levels have been observed in mouse dental papilla cells (Wang et al., 2010). WNT6 has also been found in hDPCs *in vitro* (Wang et al., 2010; Li et al., 2014). The BrdU incorporation assay and flow cytometry have shown that the percentage of proliferating hDPCs in the WNT6 protein expression group is similar to that of the control group, indicating that WNT6 protein does not promote cell cycle progression in hDPCs. Thus, WNT6 does not affect the proliferation of hDPCs. However, WNT6 increases the number and size of calcified nodules in hDPCs during the tooth mineralization process, and upregulates expression of the alkaline phosphatase gene and dentin matrix protein 1 (DMP-1), which are important markers of biomineralization (Wang et al., 2010; Li et al., 2014). It also increases the expression of osteogenic marker genes such as osteonectin (ON) and osteopontin (OPN). Moreover, *wnt6* mRNA increases the expression of type I collagen (Col I), an extracellular matrix protein in the connective tissue of dentin that is primarily released by fibroblasts and osteoblasts (Dalgleish, 1997). WNT6 protein also activates and upregulates phosphorylated JNK and c-Jun mRNA in hDPCs to induce migration and differentiation (Li et al., 2014). These results indicate that although WNT6 is not involved in the proliferation of hDPCs, it is involved in the specific differentiation and migration of hDPCs through the β-catenin-independent WNT pathway.

### Adipogenesis and Osteoblastogenesis

Mesenchymal stem cells (MSCs) differentiate into various cell types including adipocytes and osteoblasts. The dysregulation of these two cell types strongly correlates with obesity, type 2 mellitus diabetes, and osteoporosis. Therefore, maintenance of the normal functioning of MSCs is important. WNT/β-catenin signaling is an important regulator of mesenchymal stem cells (MSCs). Cawthorn et al. (2012) found that the mRNA expression of *wnt6, wnt10a*, and *wnt10b* was markedly reduced in adipocytes both *in vitro* and in mice. The ectopic mRNA expression of *wnt6, wnt10a*, and *wnt10b* suppresses the expression of two adipogenic transcription factors, peroxisome proliferator-activated receptor-γ (PPARγ), and CCAAT/enhancer binding protein-α (C/EBPα) in 3T3-L1 pre-adipocytes and completely inhibits neutral lipid accumulation (Cawthorn et al., 2012; Guo et al., 2019), indicating that all three WNTs suppress adipogenesis, even prior to the induction of the process. By contrast, loss of the three WNTs promotes adipogenesis. Knockdown of β-catenin prevents this inhibitory event, and also increases the expression of PPARγ. Thus, β-catenin is an essential factor for the inhibition of adipogenesis (Cawthorn et al., 2012). Thus, during adipogenesis, WNT6, WNT10a, and WNT10b suppress adipogenic transcription factors to inhibit adipogenesis through the β-catenin-dependent WNT pathway. As for the upstream molecular mechanism of WNT6 during adipogenesis WNT6 is associated with C/EBPβ and lysine-specific demethylase 5A (KDM5A). C/EBPβ is a key early adipogenic factor that is expressed in 3T3-L1 pre-adipocytes. KDM5A is the transcriptional target of C/EBPβ. The overexpression of C/EBPβ and KDM5A decrease *wnt6* mRNA expression in 3T3-L1 pre-adipocytes during differentiation. Knockdown of C/EBPβ and KDM5A results in an increase in *wnt6* mRNA expression; however, KDM5A and C/EBPβ are not affected by WNT6. This suggests that KDM5A and C/EBPβ are upstream molecules of WNT6. Binding of C/EBPβ and KDM5A to the promoter of WNT6 results in its inhibition. The specific mechanism of inhibition includes the recruitment of KDM5A to the promoter of WNT6 in a C/EBPβ-dependent manner, which results in the inhibition of WNT6 transcription. Then the expression of C/EBPα and PPARγ is upregulated, which stimulates adipogenesis (Guo et al., 2019). This indicates that the inhibition of WNT6 can promote adipogenesis. Additionally, ribosomal protein S6 kinase 1 (S6K1), another molecule that stimulates adipogenesis, is activated and translocates into the nucleus to suppress the transcription of WNT6, WNT10a, and WNT10b genes by phosphorylating histone H2B (Yi et al., 2016). This indicates that the S6K1-WNT pathway is a potential therapeutic target for obesity and other related metabolic disorders. In summary, a complex network of signaling molecules is involved in the inhibition of adipogenesis by WNT6.

In addition to inhibiting adipogenesis, WNT6, WNT10a, and WNT10b also stimulate osteoblast differentiation. The ectopic expression of each *wnts* mRNA not only upregulate the expression of Twist1, a transcription factor involved in the regulation of osteoblastogenesis, but also increases the expression of alkaline phosphatase, an osteoblast marker. Moreover, they also increase the calcium content in cells, thereby promoting the differentiation of stem cells (ST cells) into osteoblasts. However, β-catenin loss prevents this stimulation. Similarly, a block by short hairpin RNA of three WNT expression suppresses osteoblastogenesis. In summary, WNT6, WNT10a, and WNT10b inhibit adipogenesis and induce osteoblastogenesis by activating the WNT/β-catenin pathway (Cawthorn et al., 2012). Although the functions of these three WNT ligands are similar, the impact of the positive expression and function of both WNT10a and WNT10b is stronger than that of WNT6 in osteoblastogenesis and adipogenesis. However, the impact of the endogenous knockdown of *wnt6* in adipogenesis and osteoblastogenesis is stronger than that of endogenous knockdown of both *wnt10a* and *wnt10b* (Vertino et al., 2005; Cawthorn et al., 2012). This indicates that the influence of WNT6 is more than that of WNT10a and WNT10b in adipogenesis and osteoblastogenesis, at least under *in vitro* conditions; however, the exact mechanism requires further studies. Collectively, WNT6, WNT10a, and WNT10b, as endogenous regulators in mesenchymal precursors, work synergistically to suppress adipogenesis and promote osteoblastogenesis, and also compensate for each other. The cooperative function of the three WNTs might be closely related to the arrangement of their genes in adjacent positions on the same chromosome.

## Diseases

### Cardiac Injury

During heart injury, several events occur to protect the heart from damage, one of which is the inhibition of WNT signaling. Inhibition of the β-catenin-dependent WNT signaling pathway decreases the infarct size during cardiac injury (Zelarayan et al., 2008; Bergmann, 2010). In adults, cardiac progenitor cells (CPCs) reside in niches and might be involved in cardiac regeneration; however, they cannot achieve functional repair of the myocardium. The secreted frizzled-related protein 2 (Sfrp2), a WNT inhibitor, is upregulated during cardiac injury and stimulates the differentiation of CPCs into cardiomyocytes in the border zone, following ischemia reperfusion injury (Kobayashi et al., 2009; Schmeckpeper et al., 2015). WNT6 protein promotes the proliferation of CPCs in mice but inhibits their differentiation via the β-catenin-dependent WNT pathway; however, Sfrp2 blocks this event. Importantly, Sfrp2 does not influence the proliferation of CPCs without the involvement of WNT6. Moreover, Sfrp2 does not have any significant effect on CPC differentiation in the absence of WNT6, indicating that the effect of proliferation suppression and differentiation promotion are a result of the interaction between WNT6 and Sfrp2. In CPCs, Sfrp2 inhibits the β-catenin-dependent pathway and activates the β-catenin-independent pathway. Moreover, it activates the β-catenin-independent JNK pathway by inhibiting the β-catenin-dependent WNT6 pathway (Schmeckpeper et al., 2015). In summary, sfrp2 combines with WNT6 to promote CPCs differentiation and inhibit proliferation, providing a theoretical basis by which WNT6 plays a role in rescuing indirect myocardial injury (Table 1).

**TABLE 1:** Summary of the expression and function of WNT6 in different diseases.

Disease	Expression	Effector(s)	Consequence(s)	References
Cardiac injury	↓	β-catenin	Proliferation, Differentiation	Schmeckpeper et al., 2015
Kidney Fibrosis	↓	TGF-β1	EMT	Beaton et al., 2016
Lung Tuberculosis	↑	Arg-1, TNF-α	Polarization	Schaale et al., 2013
		c-Myc	Proliferation	
Rett Syndrome	↑	β-catenin, IGF-1, BDNF	Rescue symptom	Hsu et al., 2020
Glioblastoma	↑	Ki-67, Cyclin D1	Proliferation	Goncalves et al., 2018
		STAT3, β-catenin	Invasion, Migration	
		–	Chemoresistance	
Colorectal Cancer	↑	Bax, caspase-3	Anti-apoptosis	Zheng and Yu, 2018
		MMP2	Migration	
Gastric Cancer	↑	β-catenin	Anti-apoptosis, chemoresistance	Yuan et al., 2013

*EMT, epithelial-mesenchymal transition; TGF-β1, transforming growth factor-β1; Arg-1, Arginase-1; TNF-α, tumor necrosis factor; IGF-1, insulin-like growth factor-1; BDNF, brain-derived neurotrophic factor; STAT3, signal transducer and activator of transcription 3; Bax, B-cell lymphoma 2-associated X protein; MMP2, matrix metallopeptidase 2.*

### Kidney Fibrosis

Studies have suggested that activation of the WNT signaling pathway is essential for the repair of renal tubular epithelium and mesangium during acute kidney injury (Terada et al., 2003; Lin et al., 2010). WNT6 expression is markedly reduced not only in diabetic nephropathy patients, but also in mice models of tubule-interstitial fibrosis (e.g., unilateral ureteral obstruction) and acute tubular injury (AKI), which are mostly caused by the deposition of extracellular matrix and the loss of epithelial integrity. Moreover, the low-expression of WNT6 protein is associated with the development of kidney fibrosis. Thus, WNT6 has a protective effect on the kidneys and prevents renal fibrosis by regulating epithelial fate. In addition, WNT6 protects the integrity of the epithelium by inhibiting transforming growth factor-β1 (TGF-β1), thereby preventing renal epithelial cells from undergoing epithelial to mesenchymal transition (EMT) through a non-canonical TGF-β1 pathway. Additionally, WNT6 also promotes *de novo* tubulogenesis under *in vitro* conditions. Renal tubular epithelial cells that express WNT6 protein form the larger spheroids and new tube-like protrusions, indicating that WNT6 promotes the formation of new kidney tubules by activating the β-catenin-dependent WNT pathway (Beaton et al., 2016). In summary, the loss of WNT6 has the potential to cause the development of renal diseases, and the expression of WNT6 induces the formation of new renal tubules, suggesting that WNT6 has regenerative and repair functions. Moreover, the ability of WNT6 expression maintain the integrity of the epithelium is also an important theoretical basis for it to become a therapeutic target for renal fibrosis.

### Lung Tuberculosis

Human tuberculosis (TB), caused by *Mycobacterium tuberculosis*, infects alveolar macrophages - the primary host cells of *M. tuberculosis*. WNT signaling reportedly has immunoregulatory functions in various inflammatory and infectious diseases such as tuberculosis and atherosclerosis (Schaale et al., 2013; Villasenor et al., 2017). Infection of the mouse lung with *M. tuberculosis* leads to an increase in the level of *wnt6* mRNA compared to other *wnt* genes. Furthermore, a positive correlation has been identified between the intensity of WNT6 protein expression, the number of *M. tuberculosis* colony forming units (CFUs), and the degree of inflammation induced by *M. tuberculosis*. A mutual relationship was identified between WNT6 expression and macrophages in TB infection. The level of *wnt6* mRNA in the macrophages of infected lung tissues depends on toll-like receptor (TLR), myeloid differentiation primary response88 (Myd88), and nuclear factor kappa (NFκB). Furthermore, *wnt6* mRNA increases the level of Arginase-1 (Arg-1) and mannose receptor 1 (MRC-1). Arg-1 is the mouse marker for alternatively activated macrophages (M2), and MRC-1 is a marker for alternative macrophage activation. These indicate that WNT6 promotes inflammation in the infected macrophages, and in uninfected macrophages by increasing anti-apoptotic factors such as B-cell lymphoma 2 (BCL-2). WNT6 changes macrophages into the M2-like phenotype by increasing Arg-1 and decreasing TNF-α (Schaale et al., 2013). Additionally, *wnt6* mRNA induces expression of the c-Myc gene in macrophages, through a β-catenin independent pathway, to promote macrophage proliferation (Varlakhanova and Knoepfler, 2009; Schaale et al., 2013). c-Myc regulates the expression of genes associated with the M2 phenotype (Pello et al., 2012); however, the WNT6/c-Myc-associated mechanism requires further study. These results suggest that WNT6 induces inflammatory responses. In summary, WNT6 is exclusively expressed in macrophages with granulomatous lesions, and promotes inflammation rather than eradicating *M. tuberculosis*. Moreover, the infection of human monocyte-derived macrophages with *M. tuberculosis* H37Rv increases *wnt6* mRNA expression, suggesting the regulation of a similar WNT homolog in human macrophages (Schaale et al., 2013).

### Rett Syndrome

Rett syndrome (RTT) is a neurological and developmental disorder caused by a mutation in the methyl-CpG-binding protein 2 (MECP2) gene. Patients usually exhibit normal development at the infant stage; however, abnormal behaviors, such as locomotion impairment, cognitive function deficits, and other intellectual disability-related symptoms appear later in life (Amir et al., 1999; Swanberg et al., 2009). MECP2, an X chromosome-linked gene, is a transcriptional repressor. *wnt6* mRNA expression is significantly reduced in MECP2 mutant mice models, controlled by MeCP2 SUMOylation (Hsu et al., 2020). MeCP2 is suppressed in multiple RTT-associated MECP2 mutations (Tai et al., 2016). Additionally, the expression of brain-derived neurotrophic factor (BDNF) and insulin-like growth factor-1 (IGF-1) improves RTT in both patients and RTT mouse models (Li et al., 2012; Li and Pozzo-Miller, 2014; Pini et al., 2014). WNT6 overexpression increases the activity of the promoters of both IGF-1 and BDNF in a dose-dependent manner in HEK293T cells, and can also restore the level of *bdnf* and *igf-1* mRNA in mouse, and suppress the binding of cAMP-responsive element binding protein (CREB) to IGF-1 and BDNF to increase the expression of two genes. WNT6 may increase MECP2 SUMOylation by increasing the expression of IGF-1 and BDNF. Thus, the overexpression of WNT6 increases the expression of both IGF-1, BDNF and MECP2 SUMOylation to partly improve abnormal behaviors. Additionally, in mutant mice, environmental enrichment (EE) also upregulates WNT6 protein expression by mediating the NMDA receptor and increases the expression of IGF-1, BDNF, and MECP2 SUMOylation. Thus, a positive regulatory loop exists between MECP2 SUMOylation and WNT6 expression, and this loop is composed of multiple molecules that improve RTT. However, the exact molecular mechanism requires further investigation. One signaling pathway might be a β-catenin-dependent WNT pathway, since the overexpression of WNT6 restores the level of β-catenin and GSK-3β phosphorylation in mutant mice. However, lentivirus-WNT6 transduction, which shows limited expressed in the amygdala, partially but not completely, rescues motor function deficits and social behavior deficits in MECP2 T158A mutant mice. The activated neuron in the amygdala is insufficient to recovery the full behavior. Therefore, it is possible that maybe other molecules, in addition to WNT6, have the potential to improve RTT (Hsu et al., 2020). Nevertheless, WNT6 signaling plays an essential role in improving RTT syndrome via the WNT/β-catenin pathway. A preliminary hypothesis is that the overexpression of WNT6 in the amygdala regulates dopamine release from the nucleus accumbens to regulate motor behavior; however, the hypothesis requires further investigation (Hsu et al., 2020). Collectively, WNT6 expression alleviates symptoms of RTT syndrome. Although one mechanism involves the β-catenin-dependent WNT pathway, several other pathways are also associated with WNT6, however, this requires further investigations.

### Glioblastoma

In adults, glioblastoma (GBM) is the most lethal tumor of the central nervous system. Grade IV glioma has the strong ability to invade and spread (Goncalves et al., 2018; Goncalves et al., 2020). Analysis of The Cancer Genome Atlas (TCGA) has shown that in low-grade glioma, *wnt6* mRNA is not overexpressed. Analysis of TCGA and other dataset of glioma tissues has shown that certain GBM patients show high expression of *wnt6* mRNA and protein in tumor cells. Analysis of various GBM subtype patients in independent queues has revealed that *wnt6* mRNA is overexpressed in several GBM molecular subtypes (classical, mesenchymal, neural and proneural) (Goncalves et al., 2018). These results suggest that the level of WNT6 expression is associated with the glioma grade; however, no significant difference has been found in the GBM subtypes. *Wnt6* mRNA expression markedly enhances the proliferation, migration, and invasion of human GBM cells through cell proliferation, as shown by enzyme-linked immunoassay, wound healing migration, and matrigel invasion assays. Furthermore, high expression of *wnt6* mRNA and protein has been shown to decrease overall survival (OS) in both intracranial mouse models and patients. Mice with WNT6-overexpressing tumors show GBM-related neurological symptoms early on. The body weight loss in WNT6 over-expressing tumors is faster than that in low expressing tumors (Goncalves et al., 2018). These results indicate that WNT6 expression is a key oncogenic and aggressive mediator of GBM. Previous reports have shown that WNT signals are closely associated with GBM resistance (Nager et al., 2012). WNT6 silenced human GBM cells are more sensitive to temozolomide (TMZ), which is a gold-standard chemotherapy drug used in GBM patients. The sensitivity of radiation treatment is not significantly affected by the level of WNT6 expression (Goncalves et al., 2018). In addition to the aforementioned functions of WNT6 in tumors, it also regulates the functions of tumor stem cells, thereby promoting tumor development. WNT6 expression is positively correlated with typical stem cell-associated genes in GBM patients, according to TCGA analysis. The levels of NESTIN and SOX2 proteins, common markers of GBM stem cells (GSCs), are significantly decreased in WNT6-silenced human GBM cells. Moreover, WNT6 increases the capacity of GBM cells to form neurospheres and the frequency of neurosphere formation (upon culturing under GSC conditions), functionally indicating that WNT6 positively correlates with the self-renewal capacity of tumor stem cells. In addition to promoting GSC form neurosphere, WNT6 also maintains the phenotype of GSCs. This indicates that WNT6 promotes the expression of glioblastoma stem cell-associated genes and functionally impacts the self-renewal capacity of the tumor (Goncalves et al., 2018).

In WNT6-silenced human GBM cells, the activation of Src family kinases (SFK), heat shock protein (HSP) family (HSP27), and the total levels of β-catenin are inhibited or decreased, indicating that WNT6 acts through the β-catenin-dependent WNT pathway, the SFK/STAT pathway, and the PI3K/AKT/mTOR pathway. However, the WNT6 target in each pathway is different in different cell lines, but the effect is the same. In the PI3K/AKT/mTOR pathway, WNT6-silenced U373 cells directly inhibit the activation of AKT and mTOR, while WNT6-silenced SNB19 cells inhibit the downstream targets such as p70 S6K and eNOS. In the SFK/STAT pathway, WNT6-silenced U373 cells increase serine 727(S727) phosphorylation to decrease signal transducer and activator of transcription 3 (STAT3) activity. WNT6-silenced SNB19 cells decrease the phosphorylation of tyrosine 705 (Y705) to decrease STAT3 activity (Goncalves et al., 2018). In GBM, WNT6 expression is regulated by Homeobox A9 (HOXA9) to stimulate the β-catenin-dependent WNT pathway. HOXA9, a transcriptional regulator, binds to the WNT6 promoter and activates the expression of WNT6 in GBM cells. Moreover, WNT6 and HOXA9 are associated with the progress of GBM, resulting in poor clinical outcome when either show a high expression level. However, the functioning of WNT6 is independent on HOXA9. Furthermore, both WNT6 and HOXA9 are co-expressed in GBM and other diseases including leukemia, testicular germ cell tumor, melanoma, and cholangiocarcinoma (Goncalves et al., 2020), indicating that the scope of WNT6 function is broader. WNT6 expression is regulated by HOXA9 and DNA methylation. In glioma, the DNA methylation level of the WNT6 promoter and specific CpG regions on the genome is associated with the expression level of WNT6. Moreover, a high level of DNA methylation of specific CpG regions in the promoter is associated with WNT6 silencing, while gene body methylation is positively associated with its expression. The CpG sites are more frequently methylated in low-grade gliomas (LGG) compared to GBM patients (Goncalves et al., 2020). This indicates that WNT6 DNA methylation is at least partly involved in the regulation of WNT6 expression in gliomas. In summary, WNT6 accelerates the development of GBM in various aspects; therefore, it is a critical biomarker of tumor recurrence. WNT6 is also a potential therapeutic target or prognostic marker of GBM.

### Colorectal Cancer

Colorectal cancer (CRC) is one of the most common malignant tumors worldwide. *Wnt6* mRNA expression is increased in HCT116 and SW480 human CRC cells compared to other three colon cancer cell lines (LoVo, SW620, and HT29) (Zheng and Yu, 2018; Li et al., 2019). The high expression of WNT6 increases the proliferative ability of CRC cells (human CRC lines) by accelerating the cell cycle. The loss of WNT6 protein inhibits the expression of caspase-3 and increases the expression of B-cell lymphoma 2-associated X protein (Bax) (an apoptosis-promoting member of the Bcl-2 family). WNT6 overexpression, however, reverses this effect. This indicates that WNT6 protein expression can regulate apoptosis-regulated genes to inhibit apoptosis and promotes tumor development. Moreover, the high level of WNT6 protein increases the expression of MMP2 and induces CRC cell migration. MMP2 is also involved in the breakdown of the extracellular matrix (Zheng and Yu, 2018). Therefore, WNT6 expression promotes the development and metastasis of CRC. In addition, the promoter region of WNT6 is bound by polymorphic adenoma-like protein 2 (PLAGL2) in the nucleus of CRC cells (Li et al., 2019). PLAGL2, a zinc finger protein derived from the PLAG gene family (Kas et al., 1998; Furukawa et al., 2001; Wezensky et al., 2010), is a proto-oncogene and a transcription factor. PLAGL2 combines with the WNT6 promoter and activates the β-catenin-dependent WNT signaling pathway, thereby stimulating various downstream target genes (such as MMP7, CCND1) and promoting tumor development (Li et al., 2019). The acceleration of tumor progression by WNT6 is not only reflected in the basic molecular mechanisms, but also in the clinical prognosis. Analysis of 106 patients with colorectal liver metastasis revealed that, compared to the low level of WNT6 protein expression, the overexpression of WNT6 protein shortens the 5-year OS rate following liver resection in colorectal liver metastasis. Moreover, the influence of WNT6 in patients with different risk levels is also different. Following liver resection for colorectal liver metastasis in low-risk patients, the 5-year OS rate was found to be lower in the group with high WNT6 protein expression level compared to the low WNT6 expression group. However, it was not significantly different in the high-risk patients. This might be attributed to the different characteristics of patients with different-risk levels. Since low-risk patients have the potential for long-term survival, they are more sensitive to WNT6 expression (Peng et al., 2019). However, these result require further study because the sample size has not been large enough. In summary, WNT6 plays a key role in promoting the development of CRC and is a potential therapeutic target.

### Gastric Cancer

Gastric cancer (GC) is one of the largest causes of cancer-related deaths worldwide. WNT6 expression is low in some primary human GC cell lines compared to cell lines derives from distant metastases such as MKN45, MKN7, and NCIN87. Not all GC cells express high levels of WNT6. In GC, caveolin-1 (Cav1) can regulate the expression of WNT6. Caveolin, a plasma membrane micro-domain enriched in cholesterol and sphingolipids, is an essential structural protein (Razani et al., 2002; Parton and Simons, 2007). Cav1 is associated with lipid transport, gene regulation, and signal transduction (Anderson and Jacobson, 2002; Williams and Lisanti, 2005). MKN45/RNAi cells expressing low levels of Cav1 were found to have a low level of *wnt6* mRNA expression, while MKN45/Cav1 cells overexpressing Cav1 had a high level of *wnt6* mRNA expression. Other GC cell lines (AGS and HEK293) also had the same expression profile, indicating that Cav1 can upregulate the expression of WNT6. MKN45/RNAiCav1 contributes to chemoresistance. MKN45/RNAi cells are more sensitive to anthracycline (epirubicin) than Cav1-expressed cells might by activating apoptosis. MKN45/RNAi cells with WNT6 knockdown have a similar effect as anthracycline. When Cav1 is not expressed, in the absence or presence of *wnt6* mRNA expression, cells are not significantly different regarding their sensitivity to anthracycline (epirubicin). By contrast, the overexpression of both WNT6 and Cav1 decreases cell death, indicating that Cav1 activates WNT6 to prevent tumor cell apoptosis, and induces chemoresistance through the β-catenin-dependent WNT pathway. These data suggest that WNT6 and Cav1 are potential therapeutic targets to improve chemoresistance. A study found that cells expressing WNT6 at low levels, when treated with anthracycline, expressed increasing levels of *wnt6* mRNA and protein in a time-dependent manner, by activating the WNT/β-catenin pathway. These results indicate that Cav1 and WNT6 increase chemoresistance by activating the WNT/β-catenin pathway. Moreover, anthracycline promotes the expression of WNT6 to increase chemoresistance. This mechanism differs from that of the classical multidrug resistance transporters (Yuan et al., 2013). Additionally, in primary cancer, WNT6 suppresses rapid proliferation in the early stages of tumorigenesis, and its expression can be silenced. However, in the advanced stages of GC, WNT6 is re-upregulated to protect the GC cells (Yuan et al., 2013), due to the different functions of WNT6 dominating at different times.

## Conclusion

WNT6 belongs to the family of WNT ligands and is a highly conserved molecule that plays various roles in mice and humans. Its expression and function begin from the early stage of embryonic formation, and play a role in several stages of life. Importantly, WNT6 has the ability to become a therapeutic target, because it plays an important role in human diseases. However, further studies are needed to elucidate the role of WNT6 in diseases. Current research has mainly used animal disease models and clinical studies to provide comprehensive evidence of the role of WNT6. Meanwhile, the research on the mechanisms underlying WNT6 in disease is are still limited; thus, additional investigations are needed to provide a better understanding of the biology and function of WNT6 and its potential as a therapeutic target in these diseases.

## Author Contributions

MW, QW, XD, and HZ wrote, performed the revisions, and reviewed the manuscript. CZ and YT conducted the literature research. All authors gave the final approval of the manuscript.

## Conflict of Interest

The authors declare that the research was conducted in the absence of any commercial or financial relationships that could be construed as a potential conflict of interest.
